# 881. Saliva-Based, Extraction-Free PCR Testing For The Detection Of Key Respiratory Pathogens

**DOI:** 10.1093/ofid/ofad500.926

**Published:** 2023-11-27

**Authors:** Orchid M Allicock, Tzu-Yi Lin, Katherine Fajardo, Devyn Yolda-Carr, Maikel Hislop, Jianhui Wang, Denora Zuniga, William D Platt, Beth Tuohy, Chikondi Peno, Anne L Wyllie

**Affiliations:** Yale School of Public Health, New Haven, Connecticut; Yale School of Public Health, New Haven, Connecticut; Yale University, New Haven, Connecticut; Yale School of Public Health, New Haven, Connecticut; Yale School of Public Health, New Haven, Connecticut; Yale University, New Haven, Connecticut; Yale University, New Haven, Connecticut; Yale University School of Medicine, New Haven, Connecticut; Yale University, New Haven, Connecticut; Yale School of Public Health, New Haven, Connecticut; Yale School of Public Health, New Haven, Connecticut

## Abstract

**Background:**

Efforts to control and monitor transmissible infectious diseases rely on large-scale screening often impaired by logistically difficult or invasive sample collection. The use of saliva as a non-invasive sample type could alleviate bottlenecks encountered in mass testing strategies. Having extensively demonstrated saliva as a sensitive sample type for SARS-CoV-2 detection, we sought to validate the approach for other common upper respiratory tract pathogens.

**Methods:**

We modified our RNA-extraction-free SARS-CoV-2 PCR test for multiplexed detection of four additional respiratory viruses (“*SalivaDirect+*”): influenza A/B, RSV, and hMPV, and singleplex detection of pneumococcus. Stability of detection was tested after storage of saliva from pathogen-positive patients at +4°C, room temperature (∼19°C) and 30°C for 72 hours. De-identified saliva samples were collected from consenting adults ≥ 18 years of age with respiratory symptoms, requiring nasopharyngeal-swab based SARS-CoV-2 testing at Yale Health, New Haven, CT, USA. Saliva samples from individuals testing nasopharyngeal-swab negative for SARS-CoV-2 at the clinical laboratory were stored at -80°C until further processing in the research laboratory.

**Results:**

We confirmed the limit of assay detection at 4 copies/µl for each target. We confirmed pathogen detection remained stable at +4°C, room temperature and 30°C for up to 72 hours. From the symptomatic testing site, 804 nasopharyngeal swabs tested negative for SARS-CoV-2; their paired saliva samples were tested with the SalivaDirect+ assay. Of those, 17 (2.1%) tested positive for one of the viruses targeted (influenza A, n=7; RSV, n=4; hMPV, n=6). No samples tested positive for influenza B. For influenza A and RSV, detection by SalivaDirect+ was comparable to testing following RNA extraction but detection of hMPV was less sensitive, even following assay modifications. In singleplex testing, 87 (10.8%) samples tested positive for pneumococcus.

**Conclusion:**

We expanded a low-cost, open-source saliva-based PCR test for detection of other common respiratory pathogens. By testing saliva samples from symptomatic, SARS-CoV-2-negative adults, we detected common respiratory viruses which were otherwise missed in testing focused solely on SARS-CoV-2.

**Disclosures:**

**Anne L. Wyllie, PhD**, Co-Diagnostics: Board Member|Merck: Grant/Research Support|Pfizer: Advisor/Consultant|Pfizer: Grant/Research Support

